# Rituximab Treatment in Acute Disseminated Encephalomyelitis Associated with *Salmonella* Infection

**DOI:** 10.1155/2021/5570566

**Published:** 2021-04-17

**Authors:** Ebru Kacmaz, Gurkan Bozan, Kursat Bora Carman, Omer Kilic, Mehmet Ozgur Arslanoglu, Ugur Toprak, Aslı Kavaz Tufan, Coskun Yarar, Ener Cagri Dinleyici

**Affiliations:** ^1^Eskisehir Osmangazi University Faculty of Medicine, Pediatric Intensive Care Unit, Eskisehir, Turkey; ^2^Eskisehir Osmangazi University Faculty of Medicine, Department of Pediatric Neurology, Eskisehir, Turkey; ^3^Eskisehir Osmangazi University Faculty of Medicine, Pediatric Infectious Disease Unit, Eskisehir, Turkey; ^4^Eskisehir Osmangazi University Faculty of Medicine, Department of Radiology, Eskisehir, Turkey; ^5^Eskisehir Osmangazi University Faculty of Medicine, Department of Pediatric Nephrology, Eskisehir, Turkey

## Abstract

Acute disseminated encephalomyelitis (ADEM) is an inflammatory, demyelinating, and rapidly progressive disorder of the central nervous system. This condition is also known as postinfectious encephalomyelitis, and it is characterized by multifocal lesions in the brain and spinal cord with widespread neurological findings. High doses of intravenous (IV) methylprednisolone, intravenous immunoglobulin (IVIG), and plasma exchange (PLEX) treatments comprise the first-line therapy. There are limited pediatric case reports refractory to standard treatment. Here, we present the case of a 17-year-old girl diagnosed with ADEM associated with *Salmonella* infection, which was treated with rituximab.

## 1. Introduction

Pediatric central nervous system (CNS) demyelinating diseases include multiple sclerosis, neuromyelitis optica spectrum disorder, and ADEM. ADEM is a monophasic, demyelinating disease and characterized by multifocal lesions in the brain and spinal cord with widespread neurological findings following infectious disease [1]. In general, the disease is seen in childhood, and diagnosis requires clinical and brain magnetic resonance imaging (BMRI) findings [2]. ADEM has been associated with various infectious diseases, including those caused by viruses and bacteria. A causative agent of infection has been found in 74% of cases [3]. Corticosteroids, IVIG, and PLEX are the treatments of choice; however, occasionally, some cases remain unresponsive to these approaches [4]. Here, we present a 17-year-old girl who had been diagnosed with ADEM associated with *Salmonella* infection, which was treated with rituximab.

## 2. Case Report

A 17-year-old previously healthy girl presented with complaints of headache, numbness in the arms and legs, weakness, and change in consciousness. She presented with complaints of vomiting and fatigue 15 d before her history; she had had headache and vomiting for 3 d previously and no history of any illness or medication use. On physical examination, she was drowsy and had impaired cooperation orientation. Her blood pressure was 110/70 mmHg; respiratory rate, 20/min; pulse, 80/min; oxygen saturation, 96%; and body temperature, 36.5°C. Upon neurological examination, her admission Glasgow coma score (GCS) was 9, and she had hyperactive deep tendon reflexes, bilateral Babinski signs, and no signs of meningeal irritation. Laboratory test results were within normal limits. Neither intracranial hemorrhage nor space-occupying lesion was detected on brain computed tomography (BCT). The patient became unconscious and thus regressed to GCS 6, whereupon she was intubated and admitted to the Pediatric Intensive Care Unit (PICU). Cefotaxime (10 d) and acyclovir (7 d) administration was initiated to treat possible infection. Levetiracetam and phenytoin treatments were initiated after she sustained several episodes of generalized tonic-clonic convulsions. On the day of admission, cerebral arterio-venous magnetic resonance angiography results were normal. On BMRI, confluent subcortical white matter lesions were observed in bilateral frontal and parietal lobes and brain stem. Punctate and arc lile contrast enhancement was seen in these areas. High doses of IV methylprednisolone (1 g/d for 7 d) and IVIG (0.5 g/kg/d for 4 d) were concomitantly initiated. The opening pressure of the cerebrospinal fluid (CSF) increased in the lumbar puncture; CSF biochemistry values were in normal ranges, and cell microscopy and culture results were negative on the tenth day of admission. Nasal and CSF polymerase chain reaction testing was negative. Anti-toxoplasma gondii, anti-cytomegalovirus, anti-treponema pallidum western blot, anti-herpes virus 1/2, Borrelia burgdorferi, Ebstein-Barr virus, Varicella zoster antibodies, Mycoplasma pneumoniae, anti-HAV-IgM, Anti-HCV and anti-human immunodeficiency 1/2 antibodies, and Brucella agglutination test results were negative. Blood and urine cultures were also negative. The Gruber–Widal test result was positive, with a *Salmonella typhi* O antigen titer of 1/160 (control as 1/80). She developed global hypertonia, decerebrate posture, and hyperreflexia with clonus during follow-up. Given her lack of improvement with high-dose methylprednisolone and IVIG, as of the fifth day postadmission, PLEX was performed five times (i.e., once every other day); this was followed by immunoabsorption once. During PICU hospitalization, vancomycin, meropenem, and amphotericin B treatments were given due to healthcare-associated infection. Twenty-three days after admission (18 days after high-dose steroid therapy, 13 days after PLEX), given the occurrence of repetitive seizures and no improvement in mental status, a rituximab off-label protocol (375 mg/m^2^ once per week, four times; compassionate use after parent's consent and the approval by Ministry of Health) was initiated. Following the first dose, her mental and general condition improved and she was extubated. She was transferred to the ward after one month of hospitalization in the Pediatric Intensive Care Unit. During her routine follow up visit, she had no symptoms and signs; MRI findings regressed within 5 months, and no new lesions were seen in follow-up MRI scans.

## 3. Discussion

ADEM is an inflammatory, demyelinating, rapidly progressive disease; it usually involves bilateral, symmetrical, and deep and subcortical white matter of the brain parenchyma and spinal cord [5]. Most ADEM patients who receive an early diagnosis and appropriate treatment recover without sustaining neurological sequelae [1]. Given the unavailability of diagnostic testing, an ADEM diagnosis is made using the updated diagnostic criteria of the International Pediatric Multiple Sclerosis Study Group (IPMSSG) [1]. As in our patient, the clinical presentation occurs following certain systemic and neurological symptoms [6]. In terms of pathogenesis, T-cell-mediated autoimmune reaction triggered by infectious causes against the myelin protein is held responsible, especially in genetically sensitive individuals. Additionally, a subgroup of the patient population has elevated titers of antibodies that specifically target myelin oligodendrocyte glycoprotein immunoglobulin G and aquaporin 4 immunoglobulin G [7]. In our detailed laboratory examination, *Salmonella typhi* O antigen titers 1/160 and 1/80 were determined. Involvement of the CNS by *Salmonella* is a major atypical presentation in childhood. *Salmonella* has several neurological complications—including postinfectious demyelination [8]—that have been seen in only a few reported cases [9, 10]. Since the current patient's encephalopathy could not be explained by fever, postictal symptoms, or systemic illness (given her abnormal BMRI) and she did not have new clinical and BMRI findings in the first five months following disease onset, we diagnosed her with ADEM associated with *Salmonella* infection, in line with the updated diagnostic criteria of the IPMSSG [1]. While BCT results can be initially normal or nondiagnostic, changes can occur, including subcortical white matter multifocal lesions after 5–14 d. BMRI is valuable in diagnosis, as it shows the best demyelinating lesions. White matter lesions in ADEM appear to be more common, wider, and symmetrical among cases of the same age, due to similar patterns of contrast retention [5]. In our patient, t2-weighted hyperintensity was present in the pons-mesencephalic junction at the level of the left parietal and basal ganglia in the fronto-parietal white matter. Lumbar puncture for CSF analysis should be performed to rule out infection. CSF results can vary, but increased opening CSF pressure [11], lymphocytic pleocytosis with usually less than 100 cells/ml, and/or increased protein are totally normal [1]. If an infectious process cannot be directly excluded, appropriate antibacterial and antiviral therapy should be initiated. High-dose steroid is the first treatment of choice, for 3–7 d; this is followed by oral glucocorticoid according to the patient's response, continued in gradually decreasing doses over four to six weeks. If there is an absence of significant improvement, other therapeutic options—including IVIG and PLEX—should be considered [1]. We treated our patient with IVIG (2 g/kg) and PLEX five times and immunoadsorption once, after 7 d of IV pulse methylprednisolone. Because our patient showed no clinically significant improvement in mental status and her neurological examination results remained unchanged, she was thought to be resistant to standard treatment; at this point, rituximab was administered once per week for four weeks. Significant disease regression was seen with the first dose. We could not find any reported case in the literature that showed improvement after the first dose.

Rituximab is a chimeric human/murine monoclonal Ig G1-type antibody developed against CD20 surface antigens expressed on B cells. It induces apoptosis that stops pre-B cells from maturing into antibody-secreting cells. The US Food and Drug Administration approves rituximab treatment for non-Hodgkin's lymphoma, chronic lymphocytic leukemia, rheumatoid arthritis, polyangiitis granulomatosis, and microscopic polyangiitis. The off-label use of rituximab, however, has been applied to the treatment of other conditions where B cells and auto-antibodies are considered involved in the pathogenesis. It is best to reserve off-label use for cases where there is no adequate response to traditional therapy [12].

In conclusion, rituximab is an alternative in treating severe ADEM, including cases where there is continued neurological deterioration or poor response to first-line therapy. A few such pediatric and adult cases have been reported [4, 13]. We present a case of pediatric ADEM that was resistant to treatment with steroids, IVIG, and PLEX; rituximab, an alternative treatment option, rendered significant improvements in our patient's clinical findings. Nonetheless, further research into the safety and efficacy of rituximab use in pediatric ADEM is needed.
